# Enhancing the immunomodulatory osteogenic properties of Ti-Mg alloy by Mg^2+^-containing nanostructures

**DOI:** 10.1093/rb/rbae104

**Published:** 2024-08-29

**Authors:** Luxin Liang, Zhengjun Lin, Ziqing Duan, Solomon-Oshioke Agbedor, Ning Li, Ian Baker, Bing Wang, Tang Liu, Hong Wu

**Affiliations:** Department of Orthopedics, The Second Xiangya Hospital, Central South University, Changsha 410011, P. R. China; Department of Spine Surgery, The Second Xiangya Hospital, Central South University, Changsha 410083, P. R. China; Department of Orthopedics, The Second Xiangya Hospital, Central South University, Changsha 410011, P. R. China; State Key Laboratory of Powder Metallurgy, Central South University, Changsha 410083, P. R. China; State Key Laboratory of Powder Metallurgy, Central South University, Changsha 410083, P. R. China; Department of Oral and Maxillofacial Surgery, Center of Stomatology, Xiangya Hospital, Central South University, Changsha 410083, P. R. China; Thayer School of Engineering, Dartmouth College, Hanover, NH 03755-8000, USA; Department of Spine Surgery, The Second Xiangya Hospital, Central South University, Changsha 410083, P. R. China; Department of Orthopedics, The Second Xiangya Hospital, Central South University, Changsha 410011, P. R. China; State Key Laboratory of Powder Metallurgy, Central South University, Changsha 410083, P. R. China

**Keywords:** Ti-Mg alloy, hydrothermal treatment, Mg^2+^-containing nanostructures, macrophage, osteogenesis

## Abstract

Facilitating an appropriate immune response is crucial for promoting bone tissue regeneration upon biomaterial implantation. In this study, the Mg^2+^-containing nanostructures on the surface of Ti-1.25Mg alloy were prepared by a one-step hydrothermal reaction method via regulating pH value to enhance the immunomodulatory osteogenic properties of Ti-Mg alloys. In neutral (HT7) or alkaline (HT9) hydrothermal treatment (HT) solution, the size of MgTiO_3_ nanostructures formed on the surface of Ti-1.25Mg alloy is smaller than that in acidic HT solution (HT5), and lamellar Mg(OH)_2_ nanostructures are found in HT7 and HT9. In addition, the sample surface has a lower roughness and higher wettability with increasing pH value. The Mg^2+^-containing nanostructures on the Ti-1.25Mg alloy inhibited inflammatory response by promoting the polarization of M2 macrophages, thereby promoting osteogenesis in vitro. The micro-CT and histological assessment proved that the regeneration of bone defect was faster in HT7 than the Ti-1.25Mg *in vivo*. Mechanically, Mg^2+^-containing nanostructures can mediate the immune response of macrophages via upregulating integrins α5β1 and inhibiting Toll-like receptors (TLR-4), subsequently inhibiting the NF-κB signaling pathway. Overall, osteoimmunity-regulating Mg^2+^-containing nanostructures on Ti-1.25Mg present a promising biomaterial for bone repair.

## Graphical abstract

**Graphical abstract:**
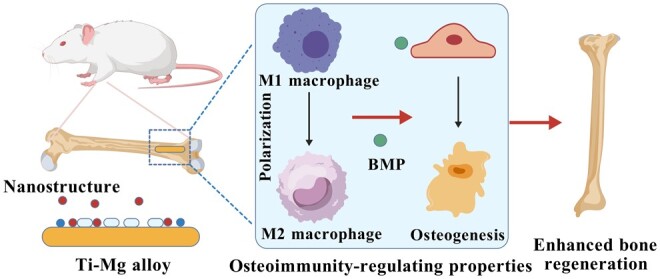

## Introduction

Bone defects caused by trauma, tumors, infection and osteoporosis are some of the most common clinical diseases [1]. Although bone tissue has the ability of spontaneous healing, the large defect will complicate bone formation. In clinical application, bone autograft and allograft as a common strategy to treat bone defects may bring many disadvantages including infection, graft rejection reaction and mismatched size [2]. It is urgently needed to develop advanced biomaterials promoting bone regeneration for the treatment of large bone defects [3]. Titanium (Ti) and its alloys are the most widely used metal materials in orthopedics due to their high strength, low density, and corrosion resistance [4, 5]. Ti and its alloys are non-toxic for cells, but could not promote the complex endogenous tissue healing, which leads to the phenomenon that the implant does not combine with natural bone [6]. So far, it has been reported that some appropriate bioactive metal ions such as Mg^2+^, Zn^2+^ and Cu^2+^ stimulate new bone formation and tissue injury repair [7–9]. Therefore, adding bioactive metal ions into Ti alloys by surface modification or alloying is an important strategy to improve osteogenesis for Ti alloy.

In our previous work, the biological metal materials containing metal magnesium (Mg) such as Ti-Mg [10, 11], Ti-Nb-Zr-Mg alloys [12], MgZnCa/Fe bulk metal glass composites [13] and ZK30/bioactive glass composites [14], show good *in vitro* biomedical performance. Especially, subsequent studies showed that the minor degradation product of Ti-Mg alloy could sequentially activate the M1 and M2 macrophage polarization, and improve the immune regulatory performance of bone at an early stage. However, higher levels of the metal Mg in Ti alloys produce severe initial inflammatory reactions and even lead to biotoxicity [6]. Qiao *et al.* [15] suggested that excessive Mg^2+^ exposure activates excessively NF-κB signaling, increases the number of osteoclast-like cells, and retards bone maturation by inhibiting hydroxyapatite precipitation in the late stage of remodeling. In addition, excessive hydroxyl ions (OH^−^), as a degradation product of magnesium, increase a local inflammatory response [16]. Prolonged inflammatory response leads to chronic inflammatory response, which is not conducive to osteogenesis. Currently, to reduce the biotoxicity caused by the rapid degradation rate of medical Mg alloys, coatings are usually prepared on the surface of Mg alloys [17, 18]. Hence, it is necessary to reduce the product of OH^−^ and excessive release of Mg^2+^ for Ti-Mg alloy.

Surface modification technologies such as hydrothermal treatment (HT), micro-arc oxidation, laser cladding, apatite coating and organic polymer coating, which can change the morphology and local composition of the surface material, are considered an effective way to improve the bioactivity of Ti alloys. Among them, the HT is one of the promising and simplest approaches for its high reactivity, low energy requirement, and easy control of the reactive system [19–21]. HT is considered to be one of the commonly used strategies to fabricate Mg^2+^-containing nanostructures on Ti [22, 23]. In previous works, the micro/nanostructures containing Ca^2+^ [24], Cu^2+^ [25] and Sr^2+^ [26] on the surface of Ti alloy were controllably fabricated by HT. More importantly, the surface physicochemical cues, such as microstructures and nanostructures, can effectively stimulate the immune response of macrophages and then regulate the osteogenic process of osteoblasts. Our previous work suggested that the microenvironment created by macrophages in response to the micro-structured coating promotes the osteogenic differentiation of SaOS-2 cells compared with the smooth Ti surface [27]. It was reported that tuning the size of the nanotubes could manipulate the macrophage polarization, which subsequently affected the *in vivo* osteogenesis and osseointegration [28, 29]. Yin *et al.* [30] reported that the anti-inflammatory microenvironment generated by M2 macrophages in response to nanostructures is more conducive to promoting osseointegration. Recently, we found that the microenvironment created by macrophages in response to the nanostructure with the size of 180 nm was more conducive to improving the osteogenic differentiation of SaOS-2 cells than that in response to the nano-structure with the size of 780 nm [24]. Therefore, we speculate that the reducing metal Mg degradation products and the increasing surface nanostructures for Ti-Mg alloy could synergistically regulate the inflammatory response, which modulates osteogenesis in the initial stage.

Several Ti-xMg (x = 0.312, 0.625, 1.25, 2.5, 5, 10, 15 wt.%) alloys were prepared using powder metallurgy [10, 11, 31]. It was found that Ti-Mg alloys improved immunomodulatory osteogenic properties compared to pure Ti [6]. In this study, Ti-1.25Mg alloy is employed as an alloy matrix model. The Mg^2+^-containing nanostructures with different sizes on the surface of Ti-1.25Mg alloy were prepared by a one-step hydrothermal reaction method via regulating pH value (acidic or alkaline). The formation mechanism of the Mg^2+^-containing nanostructures and the effect of the nanostructures on the inflammation regulation of macrophages were investigated. We further investigated the effect of the inflammatory microenvironment generated by macrophages in response to Mg^2+^-containing nanoparticles on osteogenesis *in vivo* and *in vitro*.

## Materials and methods

### Preparation of nanostructures on the surface of Ti-Mg alloy

The Ti-1.25Mg (wt.%) alloy prepared in our laboratory according to our previous work [11] was employed as raw material, and cut into discs with 15 mm in diameter and 1 mm in thickness. After being polished to 5000 mesh with abrasive paper, these discs were cleaned with acetone and ethanol for 10 min, respectively. The steps of the hydrothermal reaction are as follows. Firstly, the solutions with pH = 5, 7 and 9 were prepared by acetic acid and ammonia, respectively. Secondly, the treated discs were put into a 20 ml stainless steel reactor. Thirdly, 10 ml configured solutions were added to the reactor, respectively. Finally, stainless steel reactors were heated in an electric heating blast drying oven (DHG-9054A, China) at 200°C for 20 h. These samples were named as Ti-1.25Mg, HT5, HT7 and HT9, respectively. After being sterilized by UV, the samples were used for subsequent experiments.

### Physicochemical characterization of surface nano-structures

The scanning electron microscopy (SEM, FEI Quanta FEG250, Japan) equipped with energy-dispersive spectroscopy (EDS) was employed to observe the surface and cross-sectional morphologies. The surface roughness of various specimens was measured by atomic force microscope (AFM, Agilent 5500 AFM/SPM, USA). The water contact angles (CA) and phase composition of various surfaces were measured by utilizing an optical tensiometer (JC2000C, China) and X-ray diffractometer (XRD, Rigaku D/MAX-2250, Japan), respectively. The X-ray photoelectron spectrometer (XPS, K-ALPHA, Britain) was employed to measure the binding energy of the various elements. The transmission electron microscope (TEM, Tecnai G2 F20, USA) was employed to further analyze the phase composition and nanostructured morphology.

### Electrochemical test and ions release

The electrochemical corrosion of modified Ti-1.25Mg alloys was measured using a three-electrode set-up. A saturated calomel electrode (SCE) was used as a reference electrode, while a platinum plate served as an auxiliary electrode. The open-circuit potential (OCP) curve was tested and stabilized for 3 h in phosphate buffer solution (PBS). The potentiodynamic polarization test was measured in the range of −1.0 to 1.0 V (vs. OCP) at a scanning rate of 1 mV/s in PBS.

The experiment was carried out according to ASTM NACETM0169/G31-12a [32]. After the immersion of each specimen in PBS in an incubator at 37°C and 5% CO_2_ for 1, 3, 5 and 7 days, the inductively coupled plasma-atomic emission spectrometry (ICP-AES, Spectro blue sop, Germany) was employed to measure the concentration of ions released from the various specimens. The pH value of the solution before and after HT was measured using a pH meter (OHAUS, China).

### Viability and morphology of macrophages cultured on various surfaces

After being seeded in different material surfaces, RAW264.7 cells (Procell, China) were treated with a complete medium containing lipopolysaccharide (LPS, 100 ng/ml, Gibco) for 6 h, then the medium was replaced by complete medium for 1 and 5 days. The complete medium was prepared with 89% high glucose Dulbecco’s modified eagle medium (HG-DMEM, Thermo Fisher Scientific, USA), 10% fetal bovine serum (FBS, Gibco, USA) and 1% penicillin/streptomycin (PS, Life Technologies, USA). The fluorescence microscope (Leica, Germany) was employed to image the cells stained in 2 μM calcein-AM (Dojindo, Japan) solution. Quantitative results were obtained from the fluorescence images by utilizing Image-Pro Plus 6.0 software.

After being seeded in different material surfaces with complete medium containing LPS for 6 hours, the macrophages were cultured using complete medium for 1 day. The cells were washed with PBS, fixed with 2.5% glutaraldehyde, dehydrated in ethanol solutions with gradient concentrations, and treated with graded tertiary butanol solutions. After freeze-drying and gold-sputtering, the cells were observed under SEM (FEI Quanta FEG250, Japan). The detailed experimental methods refer to our previous literature [6].

### Flow cytometry

The flow cytometry was utilized to explore the inflammation-related protein expression of RAW264.7 cells. The CD86 and CD206 were selected as M1 and M2 markers, respectively. After being seeded in media containing LPS for 6 h, the macrophages were cultured using a complete medium without LPS for 1 and 5 days. After being incubated with FITC conjugated anti-mouse CD86 antibody (eBioscience, USA) and FITC conjugated anti-mouse CD206 antibody (eBioscience, USA), the cells were determined by a flow cytometer (Beckman, USA). The detailed experimental methods refer to our previous literature [24].

### Testing for inflammation-related genes and proteins

After being seeded with a density of 300 000 cells per well in a complete medium containing LPS for 6 h, the macrophages were cultured using a complete medium for 24 h. Real-time-quantitative polymerase chain reaction (RT-qRCR) was employed to measure the inflammation-related gene expression. The detailed experimental methods refer to our previous literature [24]. The sequences of primers used for RT-qRCR are listed in Supplementary Table S1. The concentrations of TNF-α and IL-10 in the supernatants were measured by enzyme-linked immunosorbent assay (ELISA, Cusabio Biotech, China). The detailed experimental methods refer to our previous literature [24].

### Western blot analysis

RAW264.7 cells (1 * 10^5^ cells per well) were cultured in a complete medium containing LPS for 6 h, and then the medium was replaced by a complete medium for 3 days. RAW264.7 cells were harvested and then lysed with RIPA lysis buffer. Then, proteins were separated with 10% SDS-PAGE, followed by transferring onto polyvinylidene difluoride (PVDF) membranes. Next, the PVDF membranes were blocked in the quick blocking buffer for 15 min, after which the membranes were incubated in the primary antibodies at 4°C overnight. The membranes were incubated in the corresponding secondary antibodies for 2 h. The protein bands were visualized and photographed by the chemiluminescence imaging system. The primary antibodies applied in our experiment are as follows: Tubulin (1:2000, Affinity), anti-IκBα (1:500, 340776, Zenbio, Chengdu, China), and IκBα (1:500, 380682, Zenbio, Chengdu, China).

### Evaluation of macrophage-mediated osteogenesis

The indirect co-culture method was employed to study the effect of the inflammatory micro-environments generated by RAW264.7 cells in response to different specimens on *in vitro* osteogenic differentiation. The brief experimental methods are as follows. First, the RAW 264.7 cells were cultured on different coatings for 6 h using a complete medium with LPS, and then for 24 h using a complete medium without LPS. Second, after being collected and then filtered through 0.22 μm pore-size membrane filters (Corning, USA), the supernatants were mixed with fresh McCoy’s medium (Gibco, USA) at a volume ratio of 1:6 to prepare the conditioned medium (CM) for SaOS-2. Last, SaOS-2 cells were cultured in 24-well plates with CM for 4 and 7 days to assess the alkaline phosphatase (ALP) activity and the collagen (COL) content, respectively. The detailed experimental methods refer to our previous literature [24].

### Animal surgery

Implants (Ti-1.25Mg and H7) (Φ1.5 × 8 mm) were prepared for the rat bone defect model. Our study utilized a rat femur model, and all surgical procedures were performed under sterile conditions. Rats were anesthetized by intraperitoneal injection of ketamine (10 mg kg^−1^) and 2% xylazine (5 mg kg^−1^). The rats were immobilized with the knee joint in a fully flexed position, and the right hind limb was shaved and depilated. A longitudinal incision measuring 15 mm in length was made along the lateral side of the humerus to expose the knee joint. Keeping the knee in a flexed position, a cylindrical hole with a diameter of 1.5 mm was drilled in the center of the femoral condyle, parallel to the long axis of the femur. After irrigating the bone cavity with normal saline, a metal implant was inserted into the right femur, and the wound was carefully closed. The rats were randomly assigned to two groups: (Ti-1.25Mg, *n* = 6) and (H7, *n* = 6). Post-surgery, the rats were housed in ventilated rooms with *ad libitum* access to water and food. After 4 weeks, the rats were euthanized, and the right femurs were explanted and fixed in 4% paraformaldehyde for micro-computed tomography (micro-CT) and histological analysis. All animal procedures adhered to relevant ethical regulations for animal research and were approved by the Animal Ethical Committee at the Second Xiangya Hospital (Changsha, China) (the ethical approval number: 20220671).

### micro-CT and histomorphometry analysis

About 4% paraformaldehyde-fixed rat femurs underwent an examination through micro-CT (Sky scan 1172, Bruker, Germany). After that, 3D reconstruction software (Volume Graphics, Germany) was used for visualizing the reconstructed 3D images. Region of interest (ROI) was defined as the circular bone tissue around the implants within 0.1 mm. After the micro-CT scan, hard tissue sections were prepared. The samples were first fixed with paraformaldehyde, and then rinsed with water, dehydrated with ethanol, cleaned with xylene, and finally coated with methyl methacrylate (MMA). The cutting direction is perpendicular to the long axis of the bone. Van Gieson staining and H&E staining were performed. After that, slice images were obtained using a high-resolution microscope (Olympus Co., Ltd, Tokyo, Japan). With regards to immunohistochemical staining, 4-μm-thick slices were proceeded by utilizing standard protocols. The sections were incubated with primary antibodies anti-rat CD206 (1:200), iNOS (1:200), and BMP6 (1:100) overnight at 4°C. Then the slices were incubated with the corresponding secondary antibodies for 1 h at room temperature, followed by staining with diaminobenzidine staining kit. The specimen images were observed and captured by a high-resolution microscope (Olympus Co., Ltd, Tokyo, Japan). The organ tissues of different samples were fixed, embedded, and stained with H&E to evaluate the *in vivo* biosafety of implants.

### Statistical analysis

The experimental data were from quadruplicate independent tests and shown as mean ± standard deviation (SD). The statistical significance was performed through SPSS by one-way ANOVA. The *P* < 0.05 or *P* < 0.01 was considered statistical or highly statistically significant.

## Results

### Surface physical and chemical properties

**Figure 1.**
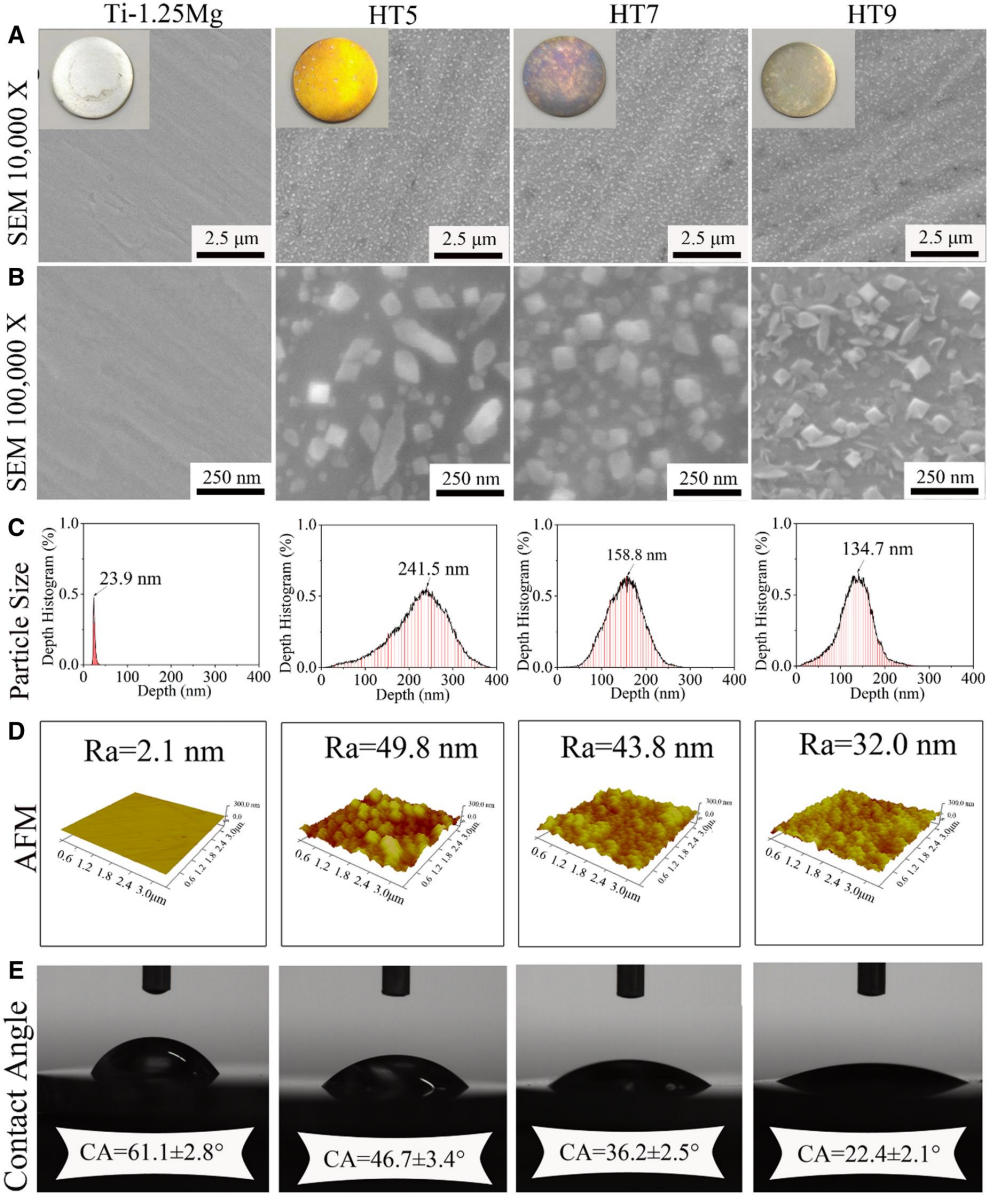
SEM (**A** and **B**), surface nanoparticles size (**C**), 3D profile and the corresponding roughness (**D**) and CA (**E**) of Ti-1.25Mg, HT5, HT7 and HT9.

As shown in Figure 1A and B, the surface color of Ti-1.25Mg, HT5, HT7 and HT9 samples shows silver white, golden yellow, purple and light yellow, respectively. At low magnification, fine nanostructures can be observed on the surface of HT5, HT7, and HT9 specimens. At high magnification, the nanostructures exhibited rod and octahedral shapes on the surface of HT5, mainly octahedral shapes on the surface of HT7, and octahedral and sheet-like shapes on the surface of HT9. The quantitative results of AFM show that the average size of nanostructure on the HT5, HT7 and HT9 surfaces was 241.5, 158.8 and 134.7 nm, respectively. The roughness of Ti-1.25Mg, HT5, HT7 and HT9 surfaces was 2.1, 49.8, 43.8 and 32.0 nm, respectively. The average wettability of Ti-1.25Mg, HT5, HT7 and HT9 surfaces was 61.1, 46.7, 36.2 and 22.4 nm, respectively. The pH values of the solution before and after the hydrothermal reaction are shown in Table 1. After the hydrothermal reaction, the PH of the solutions under either an acidic or alkaline environment tends to be neutral. These results indicated that the sample surface has a smaller nanoparticle size, lower roughness and higher wettability with increasing pH value.

**Table 1.** pH value of solution before and after HT

Samples	pH value before the reaction	pH value after the reaction
HT5	5.05	6.87
HT7	7.03	6.98
HT9	9.03	6.81

**Figure 2.**
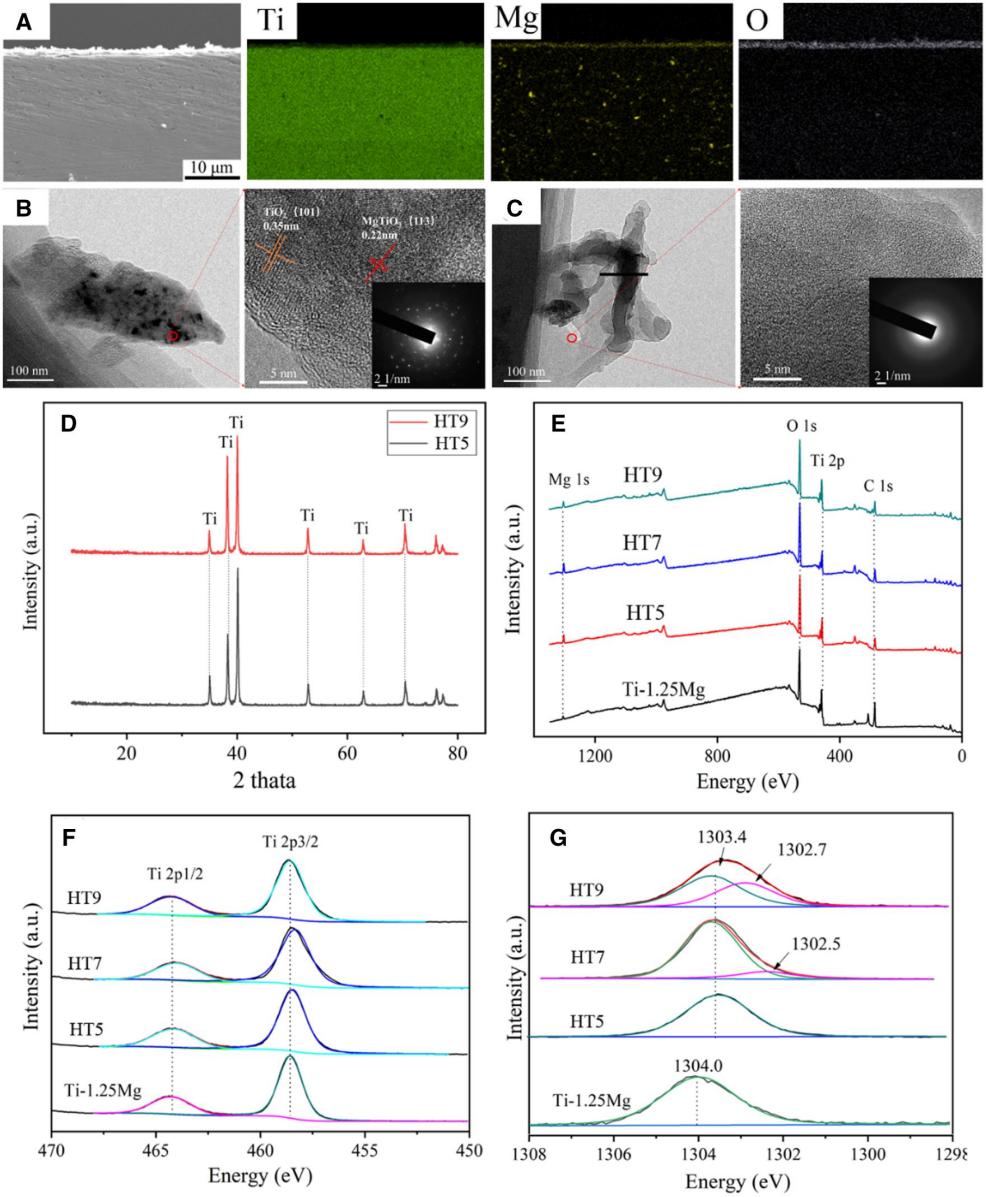
(**A**) The cross-section and EDS of HT5 sample; TEM, corresponding high-resolution and SAED pattern images of the HT5 (**B**) and HT9 (**C**); (**D**) XRD patterns of HT5 and HT9; (**E**) XPS spectrum of Ti-1.25Mg alloy, HT5, HT7 and HT9; XPS refined spectrum of (**F**) Ti 2P and (**G**) Mg1s.

To analyze the elemental composition of the surface nanostructures, the cross-section of the HT5 sample was analyzed by EDS. As shown in Figure 2A, compared with the Ti-1.25Mg alloy matrix, the surface has a lower content of Ti, while higher the content of Mg and O, indicating that compounds containing Ti, Mg and O elements were formed on the surface of the Ti-1.25Mg alloy. TEM and selected area electron diffraction (SAED) were employed to further analyze the phase of nanostructures on the surface of HT5 and HT9. As shown in Figure 2B and C, rod-like nanoparticles were composed of TiO_2_ and MgTiO_3_ phases, and flake-like nanoparticles were composed of amorphous phase. To further determine the phase composition, the XRD and XPS were employed to measure the various specimens. As shown in Figure 2D, only the Ti phase was found for HT5 and HT9 specimens, which may be attributed to the amorphous or low content of nanoparticles on the surface of specimens [11, 24]. As shown in Figure 2E–G, the Ti 2p1/2 and Ti 2p3/2 peaks at 464.3 eV and 458.5 eV indicated the presence of TiO_2_ on various surfaces [33]. The Mg 1s peak at 1304.0 eV in the Ti-1.25Mg group could be ascribed to the formation of MgO. The Mg 1s peak at 1303.4 eV in the HT5 group could be ascribed to the formation of MgTiO_3_ [33]. For the HT7 and HT9 specimens, the peak located at 1303.4 eV was ascribed to MgTiO_3_, and the peak located at 1302.5 ∼ 1302.7 eV was corresponded to Mg(OH)_2_ [33]. Together, the Mg^2+^-containing nanoparticles and TiO_2_ layer on the surface of the Ti-Mg alloy were prepared by a one-step hydrothermal reaction method via regulating pH value.

### Corrosion property and Mg^2+^ release

Supplementary Figure S1 shows the polarization curves for various specimens. Compared with the Tafel curve of the Ti-1.25Mg alloy, the curve of modified Ti-1.25Mg alloys moved in a positive potential direction. The electrode potentials of Ti-1.25Mg, HT5, HT7 and HT9 specimens were −0.5871, −0.1520, −0.2449 and −0.3398 V, respectively. The corrosion current densities of Ti-1.25Mg, HT5, HT7 and HT9 specimens were 3.8758 × 10^−6^, 1.6677 × 10^−7^, 1.0245 × 10^−6^ and 9.5644 × 10^−7^ μA/cm^2^, respectively. The corrosion rates of Ti-1.25Mg, HT5, HT7 and HT9 specimens were 0.0788, 0.0061, 0.0162 and 0.0195, respectively.

**Figure 3.**
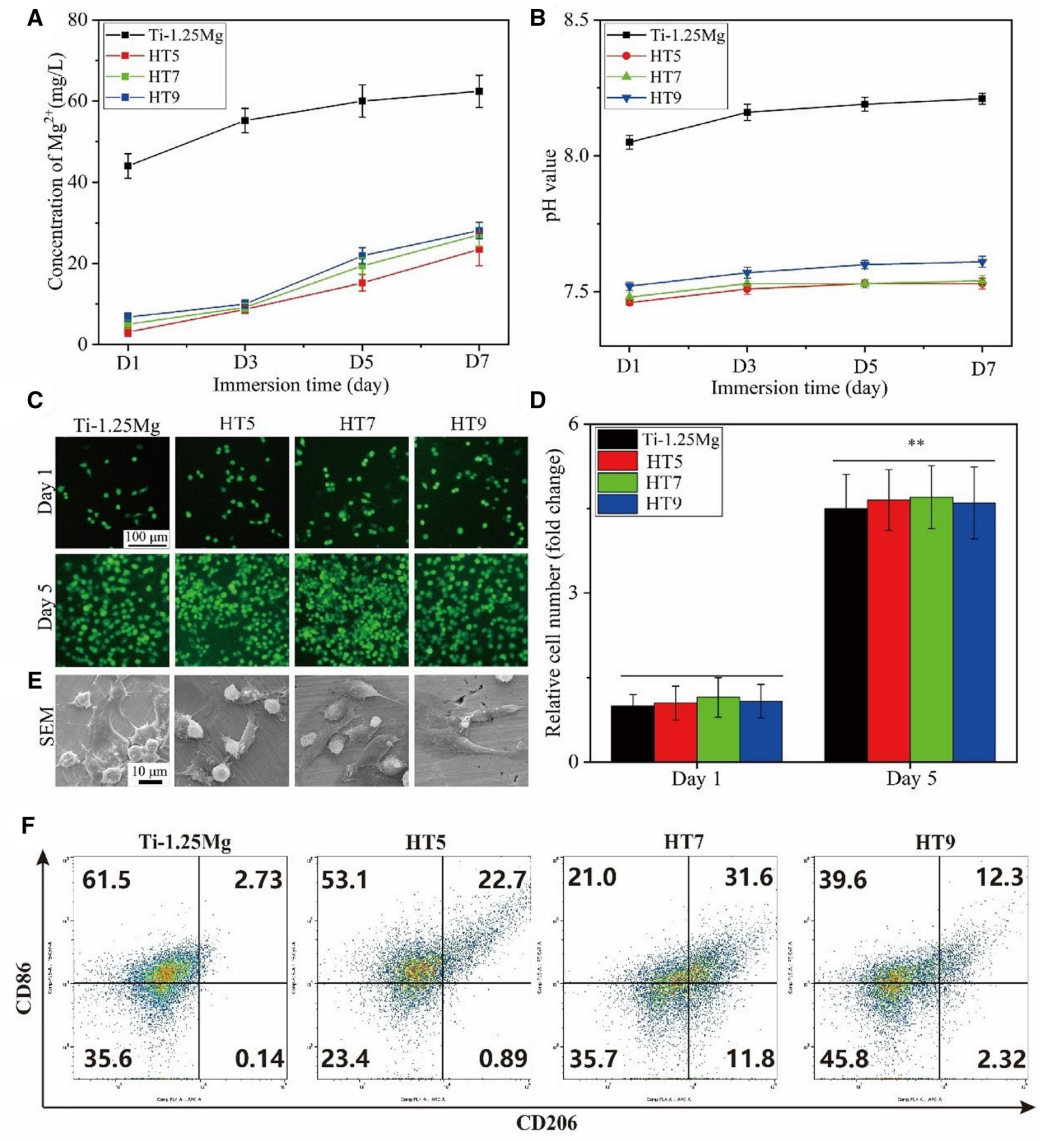
Cumulative Mg^2+^ concentration (**A**) and pH value (**B**) of the various samples released into 1 ml of PBS solution. (**C**) Live cell staining of macrophages cultured on the sample surface for 1 and 5 days, (**D**) relative quantitation of living cells, (**E**) the SEM image of cell attachment of macrophages cultured on the sample surface for 1 day. **P < 0.01 compared to the day 1 (**F**) fluorescence intensity result of CD86 and CD206 of RAW264.7 cells on various surfaces measured by flow cytometry analysis. *P < 0.05 and **P < 0.01 compared to the Ti-1.25Mg group.

As shown in Figure 3, the concentration of Mg^2+^ released from specimens after being immersed for 1, 3, 5 and 7 days was measured. The releasing curves of Mg^2+^ from various specimens were similar in shape. However, Ti-1.25Mg alloys release higher Mg ion concentrations after soaking for 1, 3, 5 and 7 days than other specimens. These results may be attributed to the fact that the Mg in the Ti-1.25Mg alloy mainly exists in the form of the metal element and MgO, while the Mg on the surface of the HT5, HT7 and HT9 exists in the compound. Several literatures reported that Mg^2+^ was slowly released when Mg^2+^ was present in the coating in the form of compounds [23, 34]. The concentration of Mg^2+^ released from the HT9 samples was higher than that from the HT5. The reason may be that the degradation rate of amorphous Mg(OH)_2_ is higher than that of crystalline MgTiO_3_ [35]. There was no significant difference between the Mg^2+^ concentration released by HT7 and HT9. This result is attributed to the fact that the Mg(OH)_2_ on the surface of HT7 and HT9 reacts chemically with the ions in the PBS solution, thus preventing the release of Mg^2+^ [36, 37].

### Macrophage proliferation and adhesion

The macrophage proliferation is shown in Figure 3C and D. The number of cells cultured on various surfaces was significantly higher on day 5 than that of cells on day 1. However, the number of macrophages on various surfaces was not significantly different on day 1 or 5. As shown in Figure 3E, macrophages present apparent differences on various surfaces. RAW 264.7 cells cultured on the surface of Ti-1.25Mg alloy showed a rounded shape. Cells cultured on the surface of HT5, HT7 and HT9 present a more elongated spindle shape as compared to the cells cultured on the surface of Ti-1.25Mg alloy. Cells cultured on the surface of HT5 showed rounded and spindle shape. The cells grown on the HT9 surface showed a more elongated spindle shape than those on the HT5 and HT7 surfaces. These results revealed that the Mg^2+^-containing nanostructures could modulate the cells shapes.

### Inflammatory response of macrophages

The flow cytometry was used to measure the proportion of M1 or M2 phenotype. The fluorescence intensity of M1 marker CD86 and M2 marker CD206 is shown in Figure 3F. The fluorescence intensity of CD86 in the Ti-1.25Mg group is significantly higher than that of HT5, HT7 and HT9 groups, while the fluorescence intensity of CD206 in Ti-1.25Mg group is significantly lower than that in HT5, HT7 and HT9 groups. There was no significant difference in the fluorescence intensity of CD206 or CD86 among HT5, HT7 and HT9 groups. These results indicated that the surface of HT5, HT7 and HT9 induced much less M1-like phenotype and much more M2-like phenotype than the Ti-1.25Mg surface.

**Figure 4.**
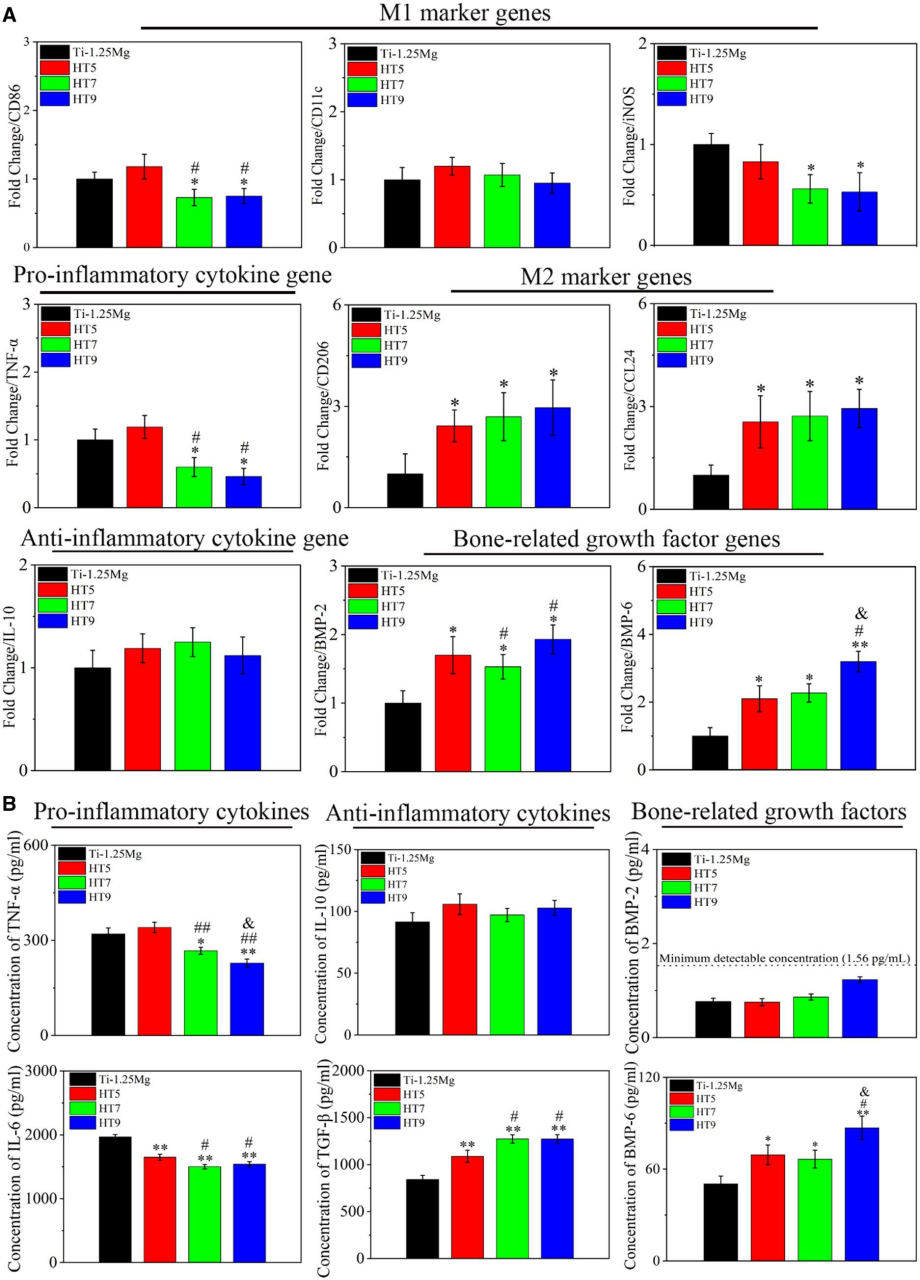
(**A**) Gene expressions of raw 264.7 cells measured by RT-qPCR. Productions of inflammatory cytokine including (**B**) TNF-α, IL-6, IL-10, TGF-β, BMP-2 and BMP-6 by macrophages cultured in complete media with different surfaces for 1 day. *P < 0.05 and **P < 0.01 compared to the Ti-1.25Mg group; #*P* < 0.05 and ##*P* < 0.01 compared to HT5; &*P* < 0.05 and &&*P* < 0.01 compared to HT7.

RT-PCR was used to measure the inflammatory-related genes in Raw264.7 cells. As shown in Figure 4A, the specimens of HT7 and HT9 inhibited the expression of pro-inflammatory genes including CD86, iNOS and TNF-α compared with the Ti-1.25Mg group. The specimens of HT5, HT7, and HT9 promoted the expression of anti-inflammatory genes (CD206 and CCL24) and growth factors (BMP-2 and BMP-6) compared with the Ti-1.25Mg group. There was no significant difference in the gene expression of IL-10 among HT5, HT7 and HT9 groups. The effect of HT5 on pro-inflammatory genes (CD86, CD11c, iNOS and TNF-α) in macrophages was not obvious at the RNA level compared with the Ti-1.25Mg group.

The protein concentration of TNF-α and IL-10 in a medium after culturing macrophages on the surface of specimens for 24 h was measured by ELISA. As in Figure 4B, the protein concentration of TNF-α in Ti-1.25Mg, HT5, HT7 and HT9 groups were 320.1, 340.8, 266.8 and 227.8 pg/ml, respectively. The protein concentration of IL-6 in Ti-1.25Mg, HT5, HT7 and HT9 groups were 1967.4, 1649.2, 1500.3 and 1540.8 pg/ml, respectively. It was suggested that the protein concentration of TNF-α and TL-6 in HT7 and HT9 groups was significantly lower than that in Ti-1.25Mg and HT5 groups. There was no significant difference in the protein concentration of IL-10 among the groups. The protein concentration of TGF-β in Ti-1.25Mg, HT5, HT7 and HT9 groups were 842.5, 1088.5, 1273.0 and 1272.3 pg/ml, respectively. At the same time, the growth factors of BMP-2 and BMP6 secreted by macrophages cultured on the surface of specimens were measured. The protein concentration of BMP-2 was below the detection range among the groups. However, the protein concentration of BMP-6 in Ti-1.25Mg, HT5, HT7 and HT9 groups were 50.4, 69.3, 66.5 and 86.9 pg/ml, respectively. The protein concentration of BMP-6 in HT5, HT7 and HT9 groups was higher than that in Ti-1.25Mg groups. In conclusion, the Mg^2+^-containing nanostructures promoted the expression of M2-related genes and proteins, while inhibiting the expression of M1-related genes and proteins.

**Figure 5.**
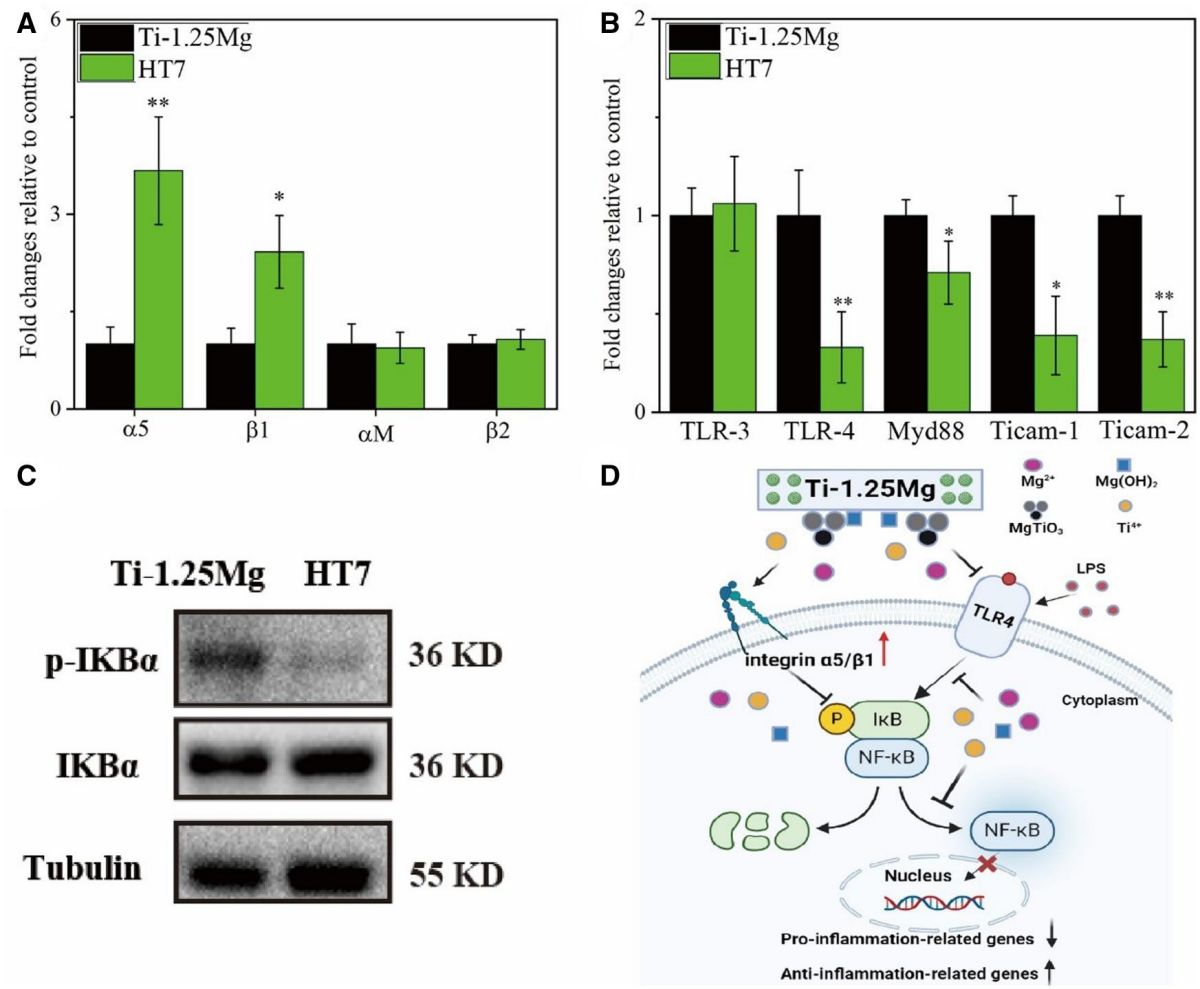
(**A**) Relative expression of integrins α5, β1, αM and β2, (**B**) relative expression of toll-like receptors (TLR-3 and TLR-4) and inflammatory signaling pathways (MyD88, Ticam1 and Ticam2). (**C**) The WB results showed the expression levels of p-IKBα and IKBα and (**D**) the pattern diagram of anti-inflammation effects of Ti-1.25Mg. *P < 0.05 and **P < 0.01 compared to the Ti-1.25Mg group.

To further analyze the underlying mechanism of inflammation in macrophages, HT7 samples were employed as the experimental group and Ti-1.25Mg as the control group to measure the related gene expression of integrin and toll-like receptor (TLR) signal pathway. As shown in Figure 5A and B, the HT7 specimen could significantly up-regulate the gene expression of integrin α5 and β1 genes in macrophages compared with the Ti-1.25Mg group. The HT7 specimen can significantly down-regulate the gene expression of TLR-4, Myd88, Ticam-2 and Ticam-2. Furthermore, LPS can stimulate the activation of the NF-κB signaling pathway to promote the expression of inflammation-related genes and the release of inflammation cytokines, thus modulating the immune response of macrophage. Therefore, we further investigated the potential functions of Mg^2+^-containing nanostructures on the Ti-Mg alloy on the NF-κB signaling pathway. Our results showed that H7 could effectively reduce the ratio of phosphorylated IκBα compared with the Ti-1.25Mg group (Figure 5C). IκBα serves as stabilizing NF-κB and binds to cytoplasmic p65 of NF-κB, allowing NF-κB to remain stable. Therefore, our results indicated that Mg^2+^-containing nanostructures on the Ti-Mg alloy can effectively suppress the NF-κB signaling pathway. The mechanistic diagram showed the inhibitory effects of the nanostructures on the Ti-1.25Mg on inflammation (Figure 5D).

### Osteogenesis in response to different osteoimmune environments

**Figure 6.**
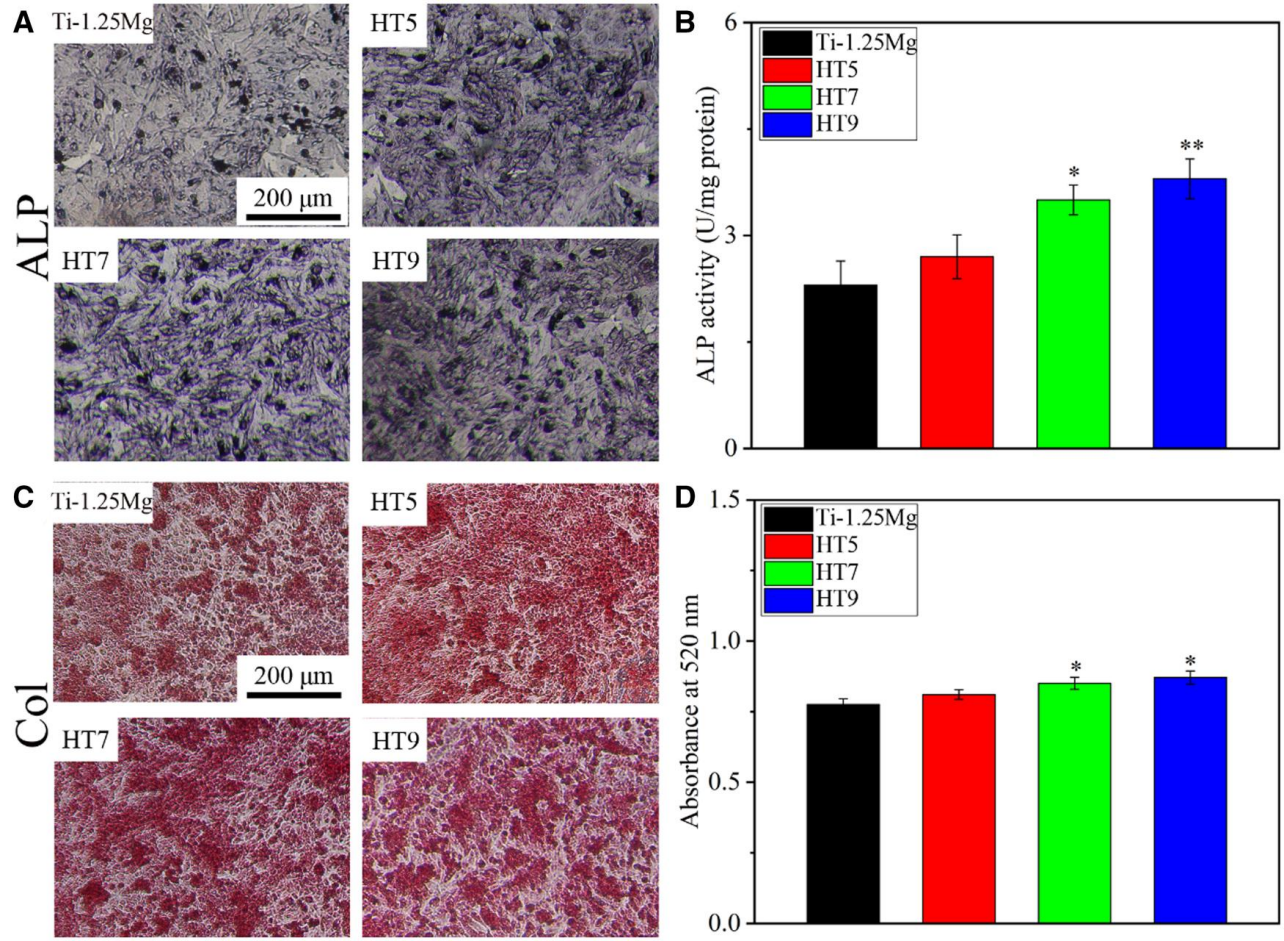
The qualitative and quantitative results of ALP activity, and col synthesis of SaOS-2 cultured by CM for 4, and 7 days, respectively. *P < 0.05 and **P < 0.01 compared to the control group.

As shown in Figure 6, the PH7 and PH9 groups promoted ALP activity for 4 days and COL synthesis for 7 days in SaOS-2 cells compared with the Ti-1.25Mg group. These results suggest that smaller nanostructures containing Mg^2+^ promoted *in vitro* osteogenesis by modulating the inflammatory microenvironment.

### *In vivo* osteoimmunity-regulating properties analysis

**Figure 7.**
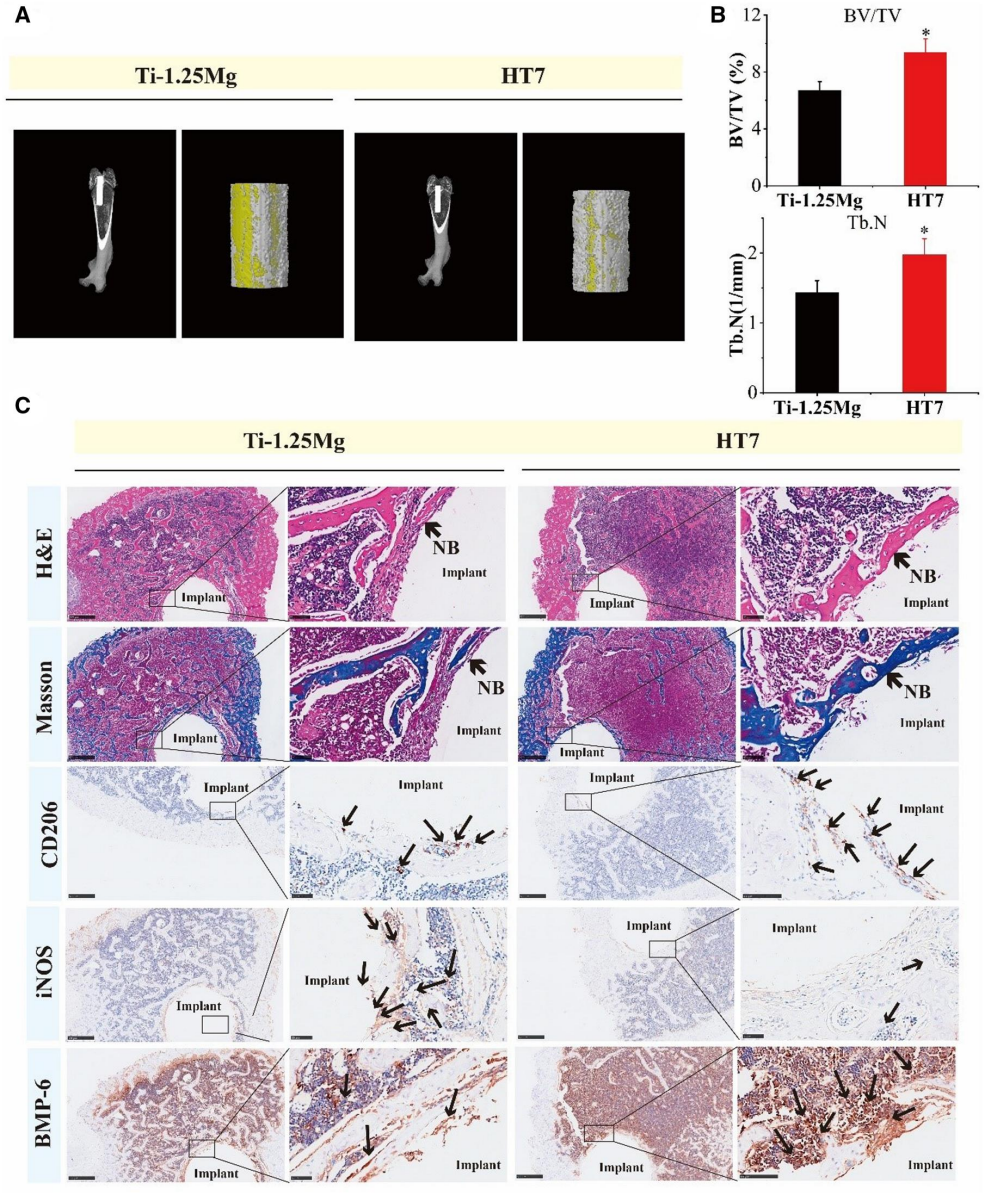
*In vivo* bone defect repair analysis of Ti-1.25Mg and HT7. (**A**) Three-dimensional and reconstructed micro-CT images. (**B**) Quantitative analyses of the osteogenesis indices. (**C**) Histological analysis results of Ti-1.25Mg and HT7, including H&E, Masson and IHC for CD206, iNOS and BMP-6. *P < 0.05 and **P < 0.01 compared to the control group.

3D Micro-CT reconstruction of HT7 and Ti-1.25Mg was used to reconstruct the femoral defect in rats (Figure 7A). Reconstructed images revealed the placement of the implants, and bone regeneration conditions *in vivo*. The image shows a small amount of new bone formation around the Ti-1.25Mg, while significantly more new bone formation and thicker new bone trabecular structures were detected in the HT7 group. Quantitative analysis results analysis showed that bone volume/tissue volume ratio (BV/TV), and Tb.N (trabecular numbers) were markedly elevated in the HT7 group, further identifying the favorable osteogenic properties of HT7 implants *in vivo* (Figure 7B).

For histomorphometry analysis, the H&E results showed that HT7 promoted the formation In Masson staining, the red color represents the host bone or newly formed bone and osteoid tissues, and the blue indicates the freshly formed bone and fibrosis tissues. Compared with the Ti-1.25Mg group, more blue tissues were detected around the HT7 group, indicating better bone ingrowth. The CD206 and iNOS immunohistochemical (IHC) staining results further confirmed the immunomodulation capacity of HT7 consistent with previous assays. The results showed that more CD206-positive cells and fewer iNOS-positive cells were found at the interface between bone and implant in the HT7 group. In addition, there were more BMP-6 positive cells in the HT7 group, further indicating the osteogenic capacity of HT7 (Figure 7C). The H&E staining of lung, liver, kidney, heart and spleen presented no abnormalities or pathological morphologies in HT7 and Ti-1.25Mg groups, suggesting that the HT7 implant had a relatively good *in vivo* biosafety (Supplementary Figure S1).

## Discussion

### Formation mechanism of nanoparticles

**Figure 8.**
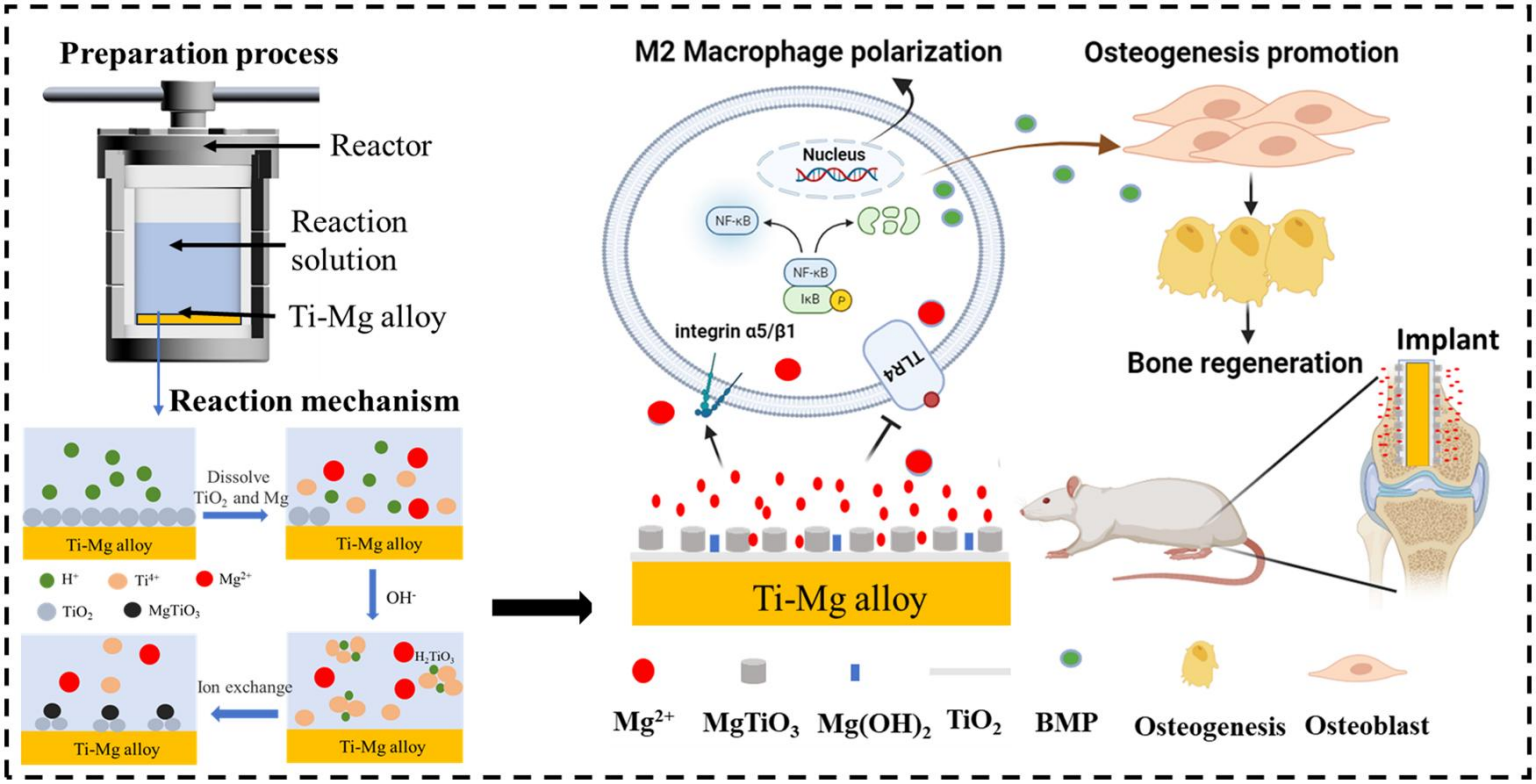
Schematic illustration of the preparation and therapeutic mechanism of Mg^2+^-containing nanostructures on the Ti-Mg alloy.

The biomimicking nanostructures on the surface of materials are considered a potential strategy to improve bone regeneration by manipulating immune cells and auto-immune environment [6, 38]. In this study, the Mg^2+^-containing nanostructures were fabricated on the surface of Ti-Mg alloy by one-step hydrothermal method. The average size of nanostructures fabricated in acidic and alkaline environments were 241.5 and 134.7 nm, respectively (Figure 1). The chemical composition of nanostructures formed in acidic environment is MgTiO_3_, while in alkaline environments are MgTiO_3_ and Mg (OH)_2_ (Figure 2). After the hydrothermal reaction, the pH of the solution under either acidic or alkaline environment tends to be neutral, while the pH of the hydrothermal solution in the neutral solution remains unchanged (Table 1). The main reason for this result may be that the Ti-Mg alloy reacted with H^+^ or OH^−^ in the solution to form nanoparticles on the surface of the alloy. The mechanism of nanoparticles formed in different environments will be discussed (Figure 8).The surface of the alloy will form an oxide film at room temperature, which is composed of TiO_2_ and MgO. When the alloy is treated in an acidic solution under high temperature and pressure, the oxide film on the surface of the alloy will react with the H^+^ to form Ti^4+^ and Mg^2+^. After a period of reaction, Ti^4+^ and Mg^2+^ were accumulated, while the H^+^ was continuously consumed to the balance between acid and alkaline in the solution. Subsequently, the Ti^4+^ reacts with Mg^2+^ to form MgTiO_3_, and the other part is dehydrated to form TiO_2_. The reaction equations are as follows.

(1) $${\text{TiO}}_{2}+{\;4H}^{+}\to {\text{Ti}}^{4+}+{2H}_{2}O$$

(2) $$\text{MgO}+{\;2H}^{+}\to {\text{Mg}}^{2+}+{\;H}_{2}O$$

(3) $${\text{Ti}}^{4+}+{\text{Mg}}^{2+}+{6\text{OH}}^{-}\to {\text{MgTiO}}_{3}+{3H}_{2}O$$

In an alkaline solution, Mg^2+^ reacts with OH^−^ to form Mg(OH)_2_ precipitation, while TiO_2_ reacts with OH^-^ to form [Ti(OH)_6_]^2−^. As the hydrothermal reaction continues, the H^+^ produced by hydrolysis reacts with Mg(OH)_2_ to form Mg^2+^. Subsequently, the [Ti(OH)_6_]^2−^ reacts with Mg^2+^ to form MgTiO_3_, and the other part is dehydrated to form TiO_2_. Hence, MgTiO_3_, Mg(OH)_2_ and TiO_2_ nanoparticles are formed in an alkaline solution. The formation mechanism of nanostructures is considered dissolution-precipitation [33, 39]. The reaction equations are as follows.

(4) $${\text{TiO}}_{2}+{2\text{OH}}^{-}+{2H}_{2}O\to {\left[\text{Ti}{\left(\text{OH}\right)}_{6}\right]}^{2-}$$

(5) $${\text{Mg}}^{2+}+{\left[\text{Ti}{\left(\text{OH}\right)}_{6}\right]}^{2-}\to {\text{MgTiO}}_{3}+{\;3H}_{2}O$$

### Macrophage response to Mg^2+^-containing nanostructures

Previous works reported that the nanostructure size, wetting angle, roughness and chemical composition had an important effect on macrophage inflammatory response [6, 24]. In our previous work, the Ti-Mg alloy promotes the pro-inflammatory response of macrophages one day 1 compared to pure Ti due to the OH^−^ released from Ti-Mg alloy [6]. The alkaline microenvironment could activate macrophages to pro-inflammatory phenotype via modulating the types, amounts or conformations of proteins. The macrophages on the surface of HT7 and HT9 were polarized to M2 phenotype compared with Ti-1.25Mg alloy, evidenced by flow cytometry, RT-PCR and ELISA. The underlying mechanism of Mg^2+^-containing nanostructures on Ti-1.25Mg alloy in polarizing macrophages to M2 phenotype was further elucidated. Nanostructured surfaces have emerged as effective approaches to modulate macrophage behavior through precise control over cell–surface interactions [40]. Integrins are cell surface receptors that play a crucial role in mediating cell adhesion to the extracellular matrix and in transmitting mechanical and chemical signals into cells [19]. Nanostructures on biomaterial surfaces can influence integrin-mediated responses in macrophages by providing spatial cues and altering the presentation of ligands. Integrin αMβ2 recognizes fibrinogen adsorbed on the surface of biomaterials and modulates biomaterial-mediated inflammatory responses [41]. In this study, the roughness and wetting angle of specimens showed a decreasing trend with the increase of the pH value in the hydrothermal reaction solution (Figure 2), and we illustrated the effects of roughness and wetting angle of specimens on integrins expression. Our results revealed that the HT7 specimen significantly upregulated the expression of integrin α5β1 in macrophages compared with the Ti-1.25Mg group. Integrin α5β1 binding to the RGD domain of fibronectin plays an indispensable role in regulating cell adhesion and function [42]. Lv *et al.* [38] have found that hydrophilic surfaces can upregulate the expression of integrin β1 in macrophages and polarize macrophages toward the M2 phenotype, while hydrophobic surfaces upregulate the expression of the integrin β2 and polarize macrophages toward the M1 phenotype.

The TLRs play important roles in modulating the inflammatory response of macrophages. The LPS polarizes macrophages to the M1 phenotype by upregulating the NF-κB via activating the TLRs signal pathway [43]. It was reported that wear-debris particles polarized macrophages to the M1 phenotype by upregulating TLR-4 and Myd88 signals [44]. The Mg^2+^-containing nanostructures on Ti-1.25Mg alloy down-regulated the gene expression of TLR-4 compared with Ti-1.25Mg alloy. The MyD88 and Ticam-1/2 are the downstream pathways of the TLR-4 signal. Akira *et al.* [45] reported that the Myd88 and Ticam-1/2 were activated by up-regulating TLR-4, which further activated the NF-κB signaling pathway, thereby releasing a large number of pro-inflammatory cytokines. In this study, the gene expression of Myd88 and Ticam-1/2 in macrophages on the surface of Mg^2+^-containing nanostructures with a size of 158.8 nm were down-regulated. Hence, the nanostructures on the Ti-1.25Mg alloy polarized macrophage to M2 phenotype via down-regulating gene expression of TLR-4, Myd88 and Ticam-1/2 compared with Ti-1.25Mg alloy. Hence, we speculated that the nanostructure cues, lower wetting angle and lower alkaline microenvironment for HT7 specimen than Ti-1.25Mg group decrease the inflammatory response of macrophage via up-regulating the integrin α5β1 and downregulating the TLR-4, Myd88, Ticam-1/2 signal axis. Furthermore, we further investigated the regulatory effects of the nanostructures on the Ti-1.25Mg alloy on the NF-κB signaling pathway, which has widely been considered a classical pro-inflammatory signaling pathway and can drive M1 macrophage polarization. When exposed to stimulation such as LPS and TNF-α, IκBα is phosphorylated, and degraded by activated IKK, subsequently resulting in the translocation of NF-κB transcription factors into the nucleus to stimulate proinflammatory biomolecules [46, 47]. In our study, HT7 was found to inhibit the phosphorylation of IκBα. The results of WB demonstrated that HT7 effectively promoted M2 macrophage polarization by hindering the NF-κB signaling pathway by stimulating integrinsα5β1 and inhibiting TLR-4 (Figure 8).

However, the HT5 specimen did not significantly reduce the inflammation of macrophages compared with Ti-1.25Mg alloy. Macrophages present roundish morphology on HT5 and Ti-1.25Mg surfaces and spindle shape on HT7 and HT9 surfaces. The polarization state of macrophages is closely related to their morphology. Numerous works reported that M0, M1 and M2 phenotypes of macrophages were spherical, roundish and spindle-shaped, respectively [16, 48, 49]. The size of nanoparticles on the surface of the HT5 specimen is larger, and the influence on macrophages is smaller than that of the HT7 or HT9 specimen. In our previous work, macrophages grown on microstructured Ti surfaces exhibited weaker anti-inflammatory properties than nano-structured Ti surfaces [26], while the pro-inflammatory properties of macrophages increased with increasing nanostructure height [24]. It was reported that the size of the nanostructures ranges from 10 to 100 nm reduces the adhesion properties and enhances the pro-inflammatory response of macrophages [50]. Hence, the large nanostructured size for HT5 samples is responsible for pro-inflammatory properties. In conclusion, Mg^2+^-containing nanostructures on HT7 and HT9 specimens mediate the immune response of macrophages via upregulating integrins α5β1 and inhibiting TLR-4, subsequently inhibiting the NF-κB signaling pathway.

### The effect of Mg^2+^-containing nanostructures on osteoimmunomodulation

Macrophages play an important role in bone formation by releasing inflammatory cytokines and osteogenic mediators [48, 51]. To explore the relationship, SaOS-2 cells were cultured using CM to determine whether factors released by macrophage response to nanostructure containing Mg^2+^ affect bone immune regulation (Figure 6). Compared with Ti-1.25Mg group, HT7 and HT9 groups enhanced ALP activity and the synthesis of COL synthesis in SaOS-2 cells, indicating that the Mg^2+^-containing nanostructures on the alloy surface promoted initial osteogenic differentiation by changing inflammatory microenvironment of macrophages. According to the results of RT-PCR and ELISA, the nanostructures on the alloy surface promoted gene expression and the secretion of protein content of BMP-6 in macrophages. These results suggest that BMP-6 released by macrophages’ response to nanostructures may be a key factor in promoting osteogenesis. Huang *et al.* [25] reported that the micro/nano morphology containing Cu^2+^ could enhance the osteogenic differentiation of SaOS-2 cells through the secretion of BMP-6 by macrophages. In our recent work, we found that bone regeneration was correlated with the content of BMP-6 secreted by macrophages in response to physical-chemical signals [6, 24]. Kemmis *et al.* [52] found that the exogenous BMP-6 in the concentration range of 50 ∼ 200 ng/ml induced osteogenesis and chondrogenesis of mesenchymal cells in mice. The protein concentration of BMP-6 in HT5, HT7 and HT9 groups was significantly higher than that in Ti-1.25Mg groups. In addition, TNF-α has a significant effect on bone healing. In the mouse model, TNF-α at a low concentration (40 μg TNF-α/kg body weight) significantly promoted fracture healing, while TNF-α at a high dose (400 μg TNF-α/kg body weight) inhibited fracture repair. In previous work, we found that although macrophages secreted a higher concentration of BMP-6 on the Micro/Nano-780 surface than on the Micro/Nano-440 surface, the enhanced production of TNF-α for the former compromised their osteogenic potential compared with the latter [24]. The protein concentration of TNF-α in Ti-1.25Mg and HT5 groups was significantly higher than that in HT7 and HT9 groups. Hence, the promotion of immune osteogenesis by Mg^2+^ nanostructures may be related to the up-regulation of BMP-6 and the decrease of TNF-α (Figure 8).

### *In vivo* analysis of osteoimmunity-regulating properties of Mg^2+^-containing nanostructures

Furthermore, we investigated the regulatory effects of Mg^2+^-containing nanostructures on macrophage polarization and bone regeneration *in vivo*. HT7 was further chosen to explore the *in vivo* effects due to several reasons. First, findings from *in vitro* biological experiments showed that compared to Ti-1.25Mg alloy, HT5, HT7 and HT9 exhibited enhanced anti-inflammatory effects. More importantly, HT7 and HT9 showed higher anti-inflammatory and osteogenic activity than Ti-1.25Mg and HT5. There was no significant difference in anti-inflammatory and osteogenic activity between HT7 and HT9. Second, the chemical composition analysis showed that the surface nanostructures of HT7 and HT9 were composed of octahedral MgTiO_3_ and lamellar Mg(OH)_2_, while the surface nanostructures of HT5 were rod-like MgTiO_3_. Third, the nano-size, roughness, and CA on the HT7 sample surface are in the middle of that on the HT5 and HT9 sample surface. The micro-CT results further confirmed a larger amount of new bone formation and thicker new bone trabecular structures around the HT7 group compared with Ti-1.25Mg. The H&E and Masson staining results further confirmed the more freshly formed bone in the HT7 group compared with the Ti-1.25Mg group. In addition, there were more BMP-2-positive cells in the HT7 group. These findings were consistent with the *in vitro* findings and further identified the favorable osteogenic properties of Mg^2+^-containing nanostructures. The IHC staining of CD206 and iNOS results showed that there were more CD206 positive cells and fewer iNOS positive at the interface between bone and implant in the HT7 group, further confirming the immunomodulation capacity of HT7 consistent with previous assays.

## Conclusions

The Mg^2+^-containing nanostructures with different sizes and phase compositions on the surface of Ti-Mg alloy were successfully prepared by the one-step hydrothermal reaction method. In an acidic environment, the nanostructure with an average size of 241.5 nm is made up of MgTiO_3_. In an alkaline environment, the nanostructures with an average size of 134.7 nm are composed of MgTiO_3_ and Mg(OH)_2_. The concentration of Mg^2+^ released from the sample after hydrothermal reaction is significantly lower than that of Ti-1.25Mg alloy. The nanoparticle cues and the low wetting angle on the sample surface facilitated M2 macrophage polarization by stimulating integrin α5β1 and inhibiting TLR-4, subsequently suppressing the NF-κB signaling pathway. The microenvironment created by macrophages in response to the nanostructure promotes osteogenic differentiation of SaOS2 cells. The Mg^2+^-containing nanoparticles on the surface Ti alloy is a promising strategy to decrease the inflammatory response of macrophages for enhanced osteogenesis.

## Supplementary Material

rbae104_Supplementary_Data
