# Supplementary material for: Enhancing the immunomodulatory osteogenic properties of Ti-Mg alloy by Mg2+-containing nanostructures
Source: Regen Biomater. 2024 Aug 29;11:rbae104. doi: 10.1093/rb/rbae104 (PMC11453102; doi:10.1093/rb/rbae104)
Supplement: rbae104_Supplementary_Data [file rbae104_supplementary_data.docx]

Supplementary Information for

**Enhancing the immunomodulatory osteogenic properties of Ti-Mg alloy by Mg^2+^-containing nanostructures**

Luxin Liang ^a,e #^, Zhengjun Lin ^a #^, Ziqing Duan ^b^, Solomon-Oshioke Agbedor ^b^, Ning Li ^c^, Ian Baker ^d^, Bing Wang ^e,*^, Tang Liu ^a,*^, Hong Wu ^b,*^

^a^ Department of Orthopedics, The Second Xiangya Hospital, Central South University, Changsha 410011, P. R. China

^b^ State Key Laboratory of Powder Metallurgy, Central South University, Changsha 410083, P. R. China

^c^ Department of Oral and Maxillofacial Surgery, Center of Stomatology, Xiangya Hospital, Central South University, Changsha 410083, P. R. China

^d^ Thayer School of Engineering, Dartmouth College, Hanover NH 03755-8000, United States

^e^ Department of Spine Surgery, the Second Xiangya Hospital, Central South University, Changsha 410083, P. R. China

#These authors have contributed equally to this work and share the first authorship

^*^ Corresponding authors

E-mail: [liutang1204@csu.edu.cn](mailto:liutang1204@csu.edu.cn) (Tang Liu); wbxyeyy@csu.edu.cn; wuhong927@126.com (Hong Wu)

Table S1 The sequences of primers employed for macrophages

| Gene | Primer sequences |
| --- | --- |
| GAPDH | Forward: 5’-TCAGCAATGCCTCCTGCAC-3’ |
|  | Reverse: 5’-TCTGGGTGGCAGTGATGGC-3’ |
| CD86 | Forward: 5’-CTGCTCATCATTGTATGTCAC-3’ |
|  | Reverse: 5’-ACTGCCTTCACTCTGCATTTG-3’ |
| CD11c | Forward: 5’-ACTTCACGGCCTCTCTTCC-3’ |
|  | Reverse: 5’-CACCAGGGTCTTCAAGTCTG-3’ |
| iNOS | Forward: 5’-CAGAAGTGCAAAGTCTCAGACAT-3’ |
|  | Reverse: 5’-GTCATCTTGTATTGTTGGGCT-3’ |
| CCL24 | Forward: 5’-AGGCAGGGGTCATCTTCATCAC-3’ |
|  | Reverse: 5’-GGCTGGTTTTTCTTGGCATCC-3’ |
| CD206 | Forward: 5’-AGACGAAATCCCTGCTACTG-3’ |
|  | Reverse: 5’-CACCCATTCGAAGGCATTC-3’ |
| TNF-α | Forward: 5’-CTGAACTTCGGGGTGATCGG-3’ |
|  | Reverse: 5’-GGCTTGTCACTCGAATTTTGAGA-3’ |
| IL-10 | Forward: 5’-TGCTATGCTGCCTGCTCTTA-3’ |
|  | Reverse: 5’-TGTTGTCCAGCTGGTCCTTT-3’ |
| BMP-2 | Forward: 5’-GCTCCACAAACGAGAAAAGC-3’ |
|  | Reverse: 5’-AGCAAGGGGAAAAGGACACT-3’ |
| BMP-6 | Forward: 5’-TGGCAGGACTGGATCATTGC-3’ |
|  | Reverse: 5’-ACCAAGGTCTGTACAATGGCG-3’ |
| α5 | Forward: 5’-CTTCTCCGTGGAGTTTTACCG-3’ |
|  | Reverse: 5’-GCTGTCAAATTGAATGGTGGTG-3’ |
| αM | Forward: 5’-CCATGACCTTCCAAGAGAATGC-3’ |
|  | Reverse: 5’-ACCGGCTTGTGCTGTAGTC-3’ |
| β1 | Forward: 5’-CGTGGTTGCCGGAATTGTTC-3’ |
|  | Reverse: 5’-ACCAGCTTTACGTCCATAGTTTG-3’ |
| β2 | Forward: 5’-CAGGAATGCACCAAGTACAAAGT-3’ |
|  | Reverse: 5’-GTCACAGCGCAAGGAGTCA-3’ |
| TLR-3 | Forward: 5’-CGGGATTGGTGAGTCTGAAG-3’ |
|  | Reverse: 5’-CGGAAAGGTGAAGGGGAG-3’ |
| TLR-4 | Forward: 5’-CACCTGATACTTGCTGGC-3’ |
|  | Reverse: 5’-TCCCCAGAGGATTGTCCTC-3’ |
| Myd88 | Forward: 5’-AGGTAAGCAGCAGAACCAGG-3’ |
|  | Reverse: 5’-TGTCCTAGGGGGTCATCAAGG-3’ |
| Ticam-1 | Forward: 5’-AGATGGTTCAGCTGGGTGTC-3’ |
|  | Reverse: 5’-TGGAGTCTCAAGAAGGGGTTC-3’ |
| Ticam-2 | Forward: 5’-CTTGGCGCTGCAAACCATC-3’ |
|  | Reverse: 5’-GCCTCTCAAATACAGACTCCCG-3’ |





Fig. S1. Tafel curves of various specimens.

Table S2. Electrochemical data determined from electrochemical experiments

| Specimens | E_corr_ (V) | i_co_*_rr_* (μA/cm2) | Corrosion rate (mm/year) |
| --- | --- | --- | --- |
| Ti-1.25Mg | -0.5871 | 3.8758×10^-6^ | 0.0788 |
| HT5 | -0.1520 | 1.6677×10^-7^ | 0.0061 |
| HT7 | -0.2449 | 1.0245×10^-6^ | 0.0162 |
| HT9 | -0.3398 | 9.5644×10^-7^ | 0.0195 |


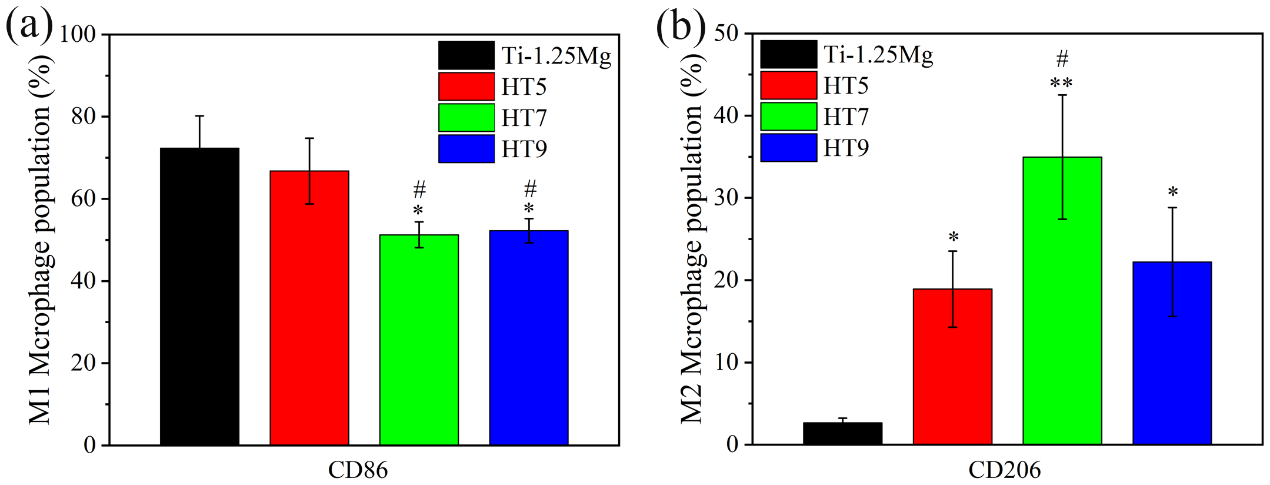


Fig.S2 Quantitative results of M1 and M2 phenotypes polarized by macrophages response to various specimens, *p<0.05 and **p<0.01 compared to Ti-1.25Mg; #p<0.05 and ##p<0.01 compared to HT5


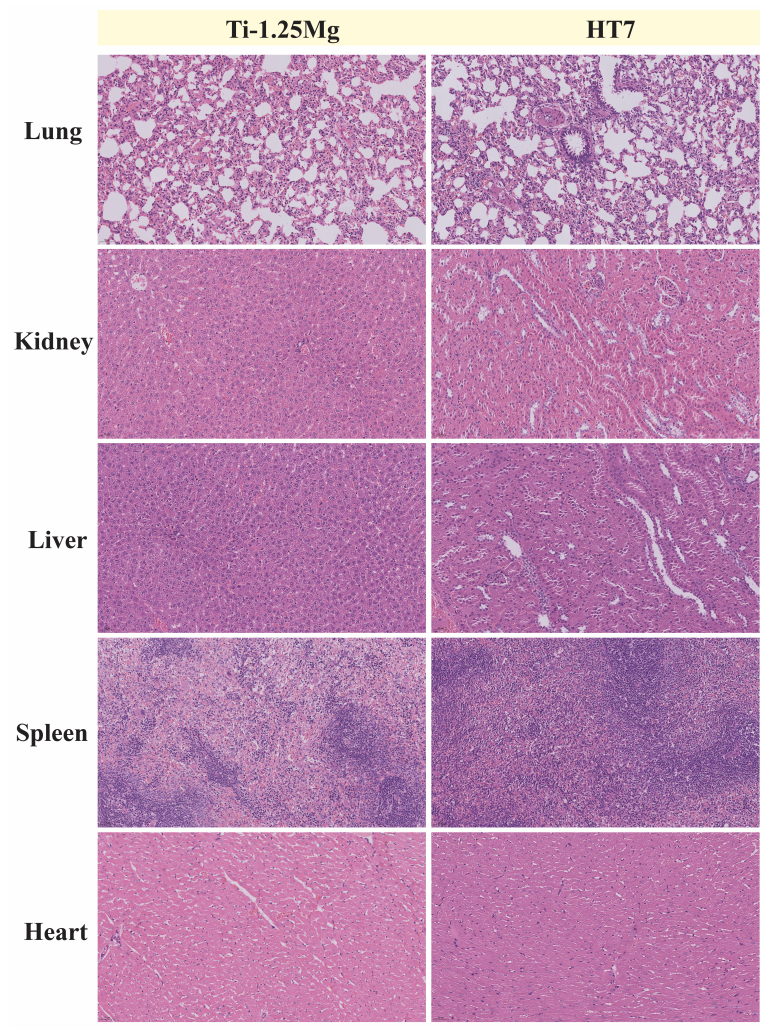


Fig. S3 The H&E staining of sampled organ tissues
